# Real-Time Chest Compression Quality Measurements by Smartphone Camera

**DOI:** 10.1155/2018/6241856

**Published:** 2018-10-28

**Authors:** Øyvind Meinich-Bache, Kjersti Engan, Tonje Søraas Birkenes, Helge Myklebust

**Affiliations:** ^1^University of Stavanger, Kjell Arholmsgate 41, 4036 Stavanger, Norway; ^2^Laerdal Medical, Tanke Svilands Gate 30, 4002 Stavanger, Norway

## Abstract

Out-of-hospital cardiac arrest (OHCA) is recognized as a global mortality challenge, and digital strategies could contribute to increase the chance of survival. In this paper, we investigate if cardiopulmonary resuscitation (CPR) quality measurement using smartphone video analysis in real-time is feasible for a range of conditions. With the use of a web-connected smartphone application which utilizes the smartphone camera, we detect inactivity and chest compressions and measure chest compression rate with real-time feedback to both the caller who performs chest compressions and over the web to the dispatcher who coaches the caller on chest compressions. The application estimates compression rate with 0.5 s update interval, time to first stable compression rate (TFSCR), active compression time (TC), hands-off time (TWC), average compression rate (ACR), and total number of compressions (NC). Four experiments were performed to test the accuracy of the calculated chest compression rate under different conditions, and a fifth experiment was done to test the accuracy of the CPR summary parameters TFSCR, TC, TWC, ACR, and NC. Average compression rate detection error was 2.7 compressions per minute (±5.0 cpm), the calculated chest compression rate was within ±10 cpm in 98% (±5.5) of the time, and the average error of the summary CPR parameters was 4.5% (±3.6). The results show that real-time chest compression quality measurement by smartphone camera in simulated cardiac arrest is feasible under the conditions tested.

## 1. Introduction

With a yearly number of out-of-hospital cardiac arrest (OHCA) incidents around 370,000-740,000 in Europe alone, and a low average survival rate of 7.6 % [1], OHCA is recognized as a major mortality challenge [2]. The time from collapse to care is crucial and there is a high focus on low response times of emergency medical services (EMS) [3]. A majority of EMS treated OHCAs are witnessed [4], and quality cardiopulmonary resuscitation (CPR), until EMS arrives, can have positive effects on survival [5–7]. The witness is often in close relation with the patient and could experience the situation as extremely stressful [8]. Studies have shown that telephone-assisted CPR (T-CPR) has a positive effect by getting more callers to start CPR and coaching callers to provide quality CPR [9–11]. Furthermore, CPR feedback has been shown to improve CPR quality [12–15]. Combining T-CPR with CPR feedback may improve CPR quality and survival from OHCA.

In the recent statement from the America Heart Association (AHA), the use of digital strategies to improve healthcare in general and to document its effect is encouraged [16, 17]. Devices providing the *bystander* with CPR quality measurement by utilizing an accelerometer to measure CPR metrics are available [18–20]. A challenge with these devices is to get the users to carry it with them at all times. Smartwatches has a built-in accelerometer, and has been suggested as a tool for measuring CPR metric [21–23]. However, a very small percentage of the population wears a smartwatch at all times. The smartphone, on the contrary, is a digital device most people carry with them. In recent years, smartphone applications have been developed for CPR quality measurement and to support learning [24, 25] and to help communicate the location of an emergency [26]. In addition, there are publications describing the use of the accelerometer in smartphones to measure CPR metrics [25, 27–30]. Smartphone solutions utilizing the accelerometer require the smartphone to be held on the patient's chest or strapped to the bystander's arm while performing CPR. These solutions may be more suited for training than for actual emergencies since buttons causing phone connection interruptions with the emergency unit can accidentally be pressed when performing the compressions.

Our research group has earlier presented an application, *QCPR cam-app 1.0,* utilizing the smartphone camera to estimate the chest compression rate and provide feedback to both the *bystander* and the *dispatcher* while the phone is placed *flat on the ground* [31]. Besides from a small offline study by Frisch et al. [32] we have found no other published work or products that utilize the smartphone camera when measuring compression rate. *QCPR cam-app 1.0* demonstrated accuracy issues when challenged with bystanders having long loose hair and in cases of people moving around the emergency scene. In this paper, we present test results of *QCPR cam-app 2.0*, improved to handle this, but also to provide more information by calculating a CPR summary report after CPR has ended. These parameters can be used to evaluate each session and to generate data that can be used for dispatcher-caller quality improvement and research.

## 2. Materials and Methods

The application, *QCPR cam-app 2.0,* captures CPR movements utilizing the smartphone camera while the smartphone is placed flat on the ground next to the patient. From the detected motions, the algorithm estimates the chest compression rate and hands-off time and provides: (1) real-time objective feedback to the bystander, (2) real-time objective feedback to the dispatcher during the emergency call, and (3) a CPR summary report.

### 2.1. Illustration of Bystander and Dispatcher Use

**Figure 1:**
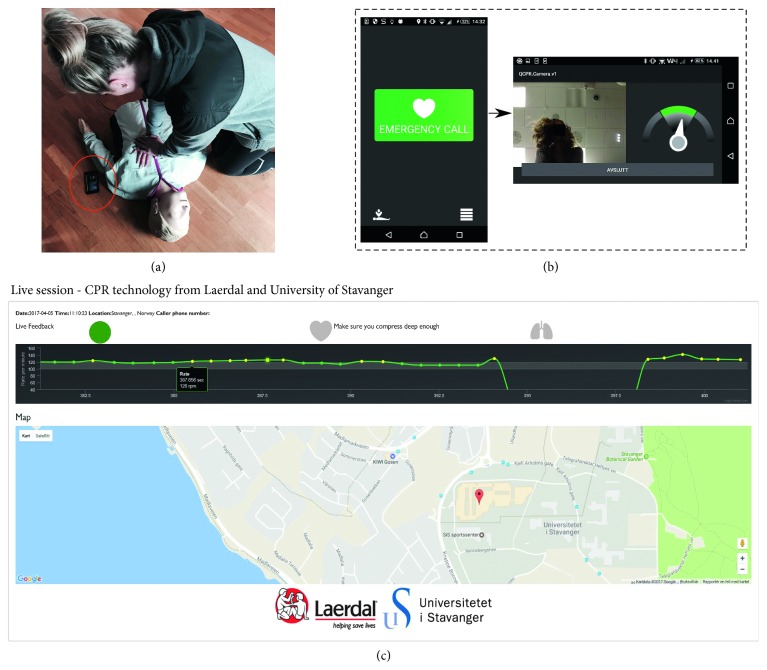
(a) Illustration photo of the smartphone application in use in a simulated emergency situation. (b) Screenshots of the smartphone application. Front page to the left and bystander feedback example to the right. (c) Screenshot of the web server available for the dispatcher.

An illustration of the application in use can be seen in Figure 1, with screenshots in Figure 1. By clicking the emergency button, the application activates speaker mode, establishes telephone connection with the dispatcher and sends GPS location and real-time compression data to a web server available for the dispatcher. The bystander then places the smartphone at the opposite side of the patient, see Figure 1. The preview frames from the front camera are shown to the bystander, allowing him to position himself and to keep track of the ongoing activity in the field of view of the camera (Figure 1). A speedometer is displayed next to the preview frame allowing the bystander to keep track of the applied compression rate.

A live sequence example of the proposed web server solution monitored by the dispatcher is shown in Figure 1. A 20 seconds sliding window providing the development and history of the compression rate in real-time is shown, where different colors are used to make the interpretation easier. Green dots correspond to compression rates in the desired range of 100–120 cpm and yellow outside. Above the graph, a circular color indicator provides information about the certainty of the reported compression rates. If the detections are carried out in low noise, the indicator is green, but if high noise conditions are present, i.e., some cases of long loose hair and from large disturbances, the indicator shifts to yellow. The bystander's GPS location is provided to the dispatcher, as seen in Figure 1.

### 2.2. Technical Description

**Figure 2:**
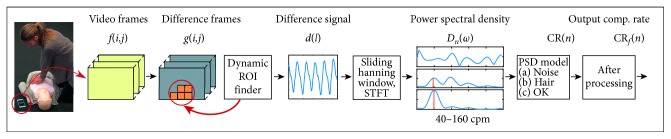
Simplified block scheme of the proposed system for chest compression rate measurement. Image frames from the smartphone front camera is used as input, and output is the detected compression rate, CR_*f*_(*n*).

*QCPR cam-app 2.0* was designed to handle the disturbance issues observed in *QCPR cam-app 1.0*, [31] and the technical description of the improvements are presented in more detail in the appendix. In short; All the estimations are performed on the smartphone, and the main steps in detection of compression rate are illustrated in Figure 2. In step 1, difference frames, *g*(*i*, *j*), are generated by thresholding the differences between subsequent input frames, *f*(*i*, *j*), from the camera. A dynamic region of interest (ROI) is established from the largest connected moving object and is updated each half second by checking the activity in the blocks around the ROI boundary. By using a dynamic ROI, we allow others to move around in the emergency scene without disturbing the detections. In step 2, we generate a signal, *d*(*l*), from the activity in the ROI and for each half second, timestep *n*, a short time Fourier transform (STFT) is performed on the three last seconds of *d*(*l*). A sliding Hanning window is applied to *d*(*l*) prior to the STFT. In step 3, the power spectrum density, *D*_*n*_(*ω*), found from the STFT is studied and a decision three is used to separate compression rates from noise. The decision three recognizes a system in the *D*_*n*_(*ω*) for cases of bystanders with long loose hair, thus solve the detection issues observed in *QCPR cam-app 1.0* [31] for these cases. If a CR(*n*) is detected, it further undergoes some postprocessing steps, indicated in step 4, Figure 2. These steps filter out and suppress noise by performing smoothing and removing short detection pauses caused by compression stops or disturbances. In step 5, the detected and filtered compression rate, CR_*f*_(*n*) (cpm), is displayed on the smartphone and sent to the web server and displayed to the dispatcher, providing the real-time feedback to both bystander and dispatcher.

### 2.3. CPR Summary Report

After completion of a caller session, a set of CPR summary parameters are calculated by *QCPR cam-app 2.0*. The parameters, which are both shown on the smartphone screen for the bystander and saved on the web server for the dispatcher, are as follows:

- *TFSCR (s)*: time from start of phone call to start of first stable compression rate. A compression rate is defined as stable if CR_*f*_(*n*) > 40 and |CR_*f*_(*n*) − CR_*f*_(*n* − 1)| < 20 is true for at least 6 seconds.
- *TC (s)*: total active compression time. The time where CR_*f*_(*n*) > 0, for *t*(*n*) > TFSCR, and continuously for more than 2 s.
- *TWC (s)*: time without compressions. *TDPC-TC*, where *TDPC (s)* is the duration of the phone call.
- *ACR (min* ^*−1*^): average compression rate. An average of all CR_*f*_(*n*) > 0, for *t*(*n*) > TFSCR, and continuously for more than 2 s.
- *NC*: total number of compressions. Estimated by: *ACR* ^*∗*^ (*TC/60*).

### 2.4. Data Material and Evaluation Measures

All experiments were performed on a Resusci Anne QCPR manikin. The *QCPR cam-app 2.0* algorithm was implemented in Android Studio and the experiments were performed with a Sony Xperia Z5 Compact (Sony, Japan). A reference signal for the compression rate was provided by an optical encoder embedded in the Resusci Anne QCPR. A three-second long sliding window frequency analysis was performed on the signal each half second, providing the reference data, CR_true_(*n*), with the same sample rate as the compression rate detection, CR_*f*_(*n*), from the app.

To evaluate the results, different measurements were used: *Average error* ($\bar{E}$), *Performance* (*P*), *Relative error parameter* (RE_par_) and *Bland Altman* plots used to visualize the agreement between data provided by *QCPR cam-app 2.0* and the reference data provided by Resusci Anne QCPR manikin. $\bar{E}$ is given in compressions per minute (cpm) and is the average error of the sequence, defined as

(1) $$\begin{array}{c} \bar{E}\left[\text{cpm}\right]=\frac{1}{N}\overset{N}{\underset{n=0}{\sum }}\left|{\text{CR}}_{f}\left(n\right)-{\text{CR}}_{\text{true}}\left(n\right)\right|, \end{array}$$

where *N* is the number of samples of the sequence. For sequences containing discontinuity in the reference data, i.e., 30 : 2 session, we allowed errors in a ±1 s interval around the automatically detected discontinuities. This reduced the influence of insignificant delays on the error measure. *P* is defined as the percentage of time where |CR_*f*_(*n*) − CR_*true*_(*n*)|<Δ. According to guidelines [33–35] the acceptable compression rate is between 100 and 120 cpm, thus Δ *=* 10 (cpm) was chosen as an acceptance criterion. RE_par_ measures the performance of the CPR summary parameters listed in Section 2.3. RE_par_ is given in percentage and defined as

(2) $$\begin{array}{c} {\text{RE}}_{\text{par}}\left[\%\right]=\frac{\left|{\text{Par}}_{D}-{\text{Par}}_{R}\right|}{{\text{Par}}_{R}}100, \end{array}$$

where Par_*D*_ is a CPR summary parameter estimated by the app and Par_*R*_ the corresponding CPR parameter found from the reference signal. If the test contained more than one sequence, the results are presented with mean and standard deviations, i.e., $\mu \bar{E}\left(\sigma \bar{E}\right)$, *μP*(*σP*), and *μ*RE_par_(*σ*RE_par_), found over the result values of the sequences.

Desired detection results provide a low *Average error*, $\bar{E},$ a low *Relative error parameter*, RE_par_, and a high *Performance*, *P*.

### 2.5. Experiments

The performance of the *QCPR cam-app 2.0* was tested in various conditions that could occur in real emergencies. The experiments were divided into five different tests—*Smartphone position test, Outdoor test, Disturbance test, Random movement test*, and *CPR summary report test*. Altogether, this sums up to approximately 162 minutes of CPR. Specifications for the subtests included in each test are listed in Table 1.

The *Smartphone position test* included seven test persons—two with short hair (SH), two with medium length loose hair (MLLH) i.e., chin/shoulder length, and three with long loose hair (LLH) i.e., chest length. Each of the test persons performed 8 subtests carried out indoor.

The result for subtest *RateP1*, Table 1, was presented in Meinich-Bache et al. [36] to verify that *QCPR cam-app 2.0 is* able to estimate correct compression rate for test objects with various hair lengths and for different compression rates, which were an issue in *QCPR cam-app 1.0* [31].

The subtest *D1R110P1* included a person that walks around and behind the bystander during CPR, leaning over the patient, waving his arms, and thus causing disturbances. These results were also presented in Meinich-Bache et al. [36] to verify improvements of *QCPR cam-app 2.0* over *QCPR cam-app 1.0* where sometimes disturbances could take over the dynamic ROI [31]. The results of subtests *RateP1* and *D1R110P1* are repeated here for the reader to experience all the various tests that *QCPR cam-app 2.0* has been exposed to.

**Figure 3:**
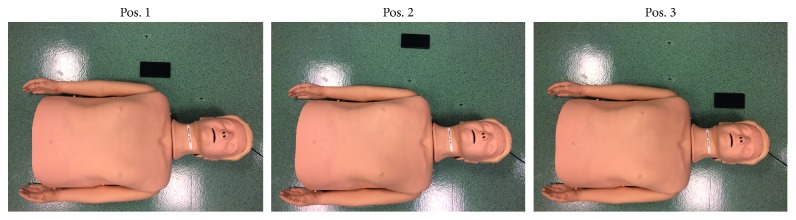
Different camera positions used in smartphone position test.

Various other conditions were also tested in the *Smartphone position test*. Three camera positions were included: *next to shoulder (Pos.1), 20 cm away from shoulder (Pos.2)*, *and next to head (Pos.3).* The camera positions are shown in Figure 3. 30 : 2 sessions were carried out for camera positions *Pos 1,* subtest *30* *:* *2P1*, and *Pos 3,* subtest *30:2P3. Pos.3* was included to see if the algorithm provides false detection when the bystander is still visible in the image frame when performing rescue breaths. Since the bystander is not visible in the image frame while performing rescue breaths when the camera is positioned in *Pos.2,* this position is not relevant for the 30:2 sessions and therefore not included. *Pos.2* is used to measure the algorithm's ability to detect when only a small part of the bystander is visible in the image frame and used in subtests *R100P2* and *R150P2.* The algorithm was also tested in low lighting conditions, 7 lux, in subtest *LightP1*.

The *Outdoor test* included three test persons, one with each hair length; SH, MLLH, and LLH. The detections were carried out in cloudy (C) and sunny (S) weather, both with and without noisy background (B) i.e., trees.

The purpose of the *Disturbance test* was two-fold: (1) to measure the algorithm's ability to detect compression rate when there is a large disturbance present i.e., another moving person, and to (2) quantify the disturbance size relative to the bystander performing the compressions when the algorithm fails to detect due to too much noise. A second Sony Xperia Z5 Compact (Sony, Japan) phone was used to capture video recordings of the test, and the video is studied offline to perform the quantification. The bystander carried out continuous compressions during the sequence. The disturbing person moved around the patient, waving arms in different frequencies, standing behind and over the bystander while waving arms, stepping over patient etc.

In the *Random movement test*, no CPR was performed on the manikin and the purpose of the test was to measure the algorithm's resilience to false detections. The random movement included checking breathing and pulse of patient, turning patient, unzipping jacket, walking around, waving for help etc. Three test persons were included.

The *CPR summary report test* is an evaluation of the session summary parameters. The test included five different test persons with different hair lengths and the following test protocols:

- The bystander sits next to patient with the smartphone in his hands. He/she presses the *emergency call* button and places the smartphone flat on the ground. For approximately 20 seconds, the bystander checks for patient's pulse and respiration before starting performing chest compressions.
- Next, four intervals of 120-second continuous compressions and 20-second pauses while checking for respiration are followed.
- The total sequence time is approximately 580 s, which is a typical response time for medical assistance [37–40].

The CPR summary parameters evaluated are the parameters explained in Section 2.3: TFSCR, TC, TWC, ACR, and NC.

## 3. Results

**Figure 4:**
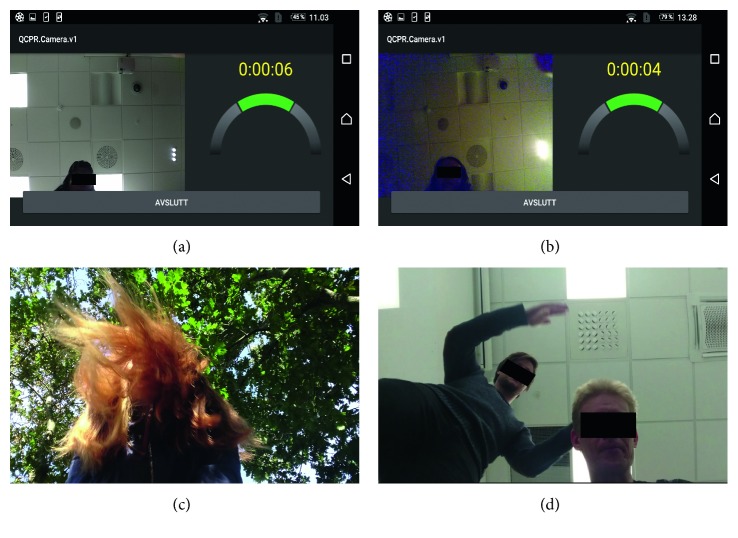
(a) Screenshot of a MLLH bystander's position in image frame when algorithm provided poor detection results for compression rate of 150 cpm, R150P2. (b) Screenshots of low lighting conditions, LightP1. (c) Screenshot of LLH and noisy outdoor background, OSBR110P1. (d) Screenshot of the disturbance size when the algorithm failed to detect the compression rate in D2R110P1.

The error measurement results of all five tests are summarized in Table 2. The average compression rate detection error, $\bar{E}$, was 2.7 compressions per minute (±5.0 cpm), the performance, *P*, accepted detections in 98% (±5.5) of the time and the relative error of the CPR summary parameters, RE_par_, were 4.5% (±3.6). In subtest *R150P2* from the *Smartphone position test,* the results reveal some weaknesses when only a small part of the bystander is visible to the camera, the compression rate is as high as 150 cpm and the person performing compression has MLLH or LLH. In the two sequences with poor results, *P* of 56.2% and 80.2%, the bystander is only present in 4.6% and 6.9% of the image frame and an example from the largest one is shown in Figure 4(a). Figures 4(b)–4(d) also show examples from the subtests: (B) low lighting conditions, *LightP1*, (C) LLH in noisy outdoor conditions, *OSBR110P1*, and (D) the smallest disturbance, occupying 3.4 times the size of the area occupied by the bystander, that cause the algorithm to fail to detect for a short period of time in *D2R110P1.*

**Figure 5:**
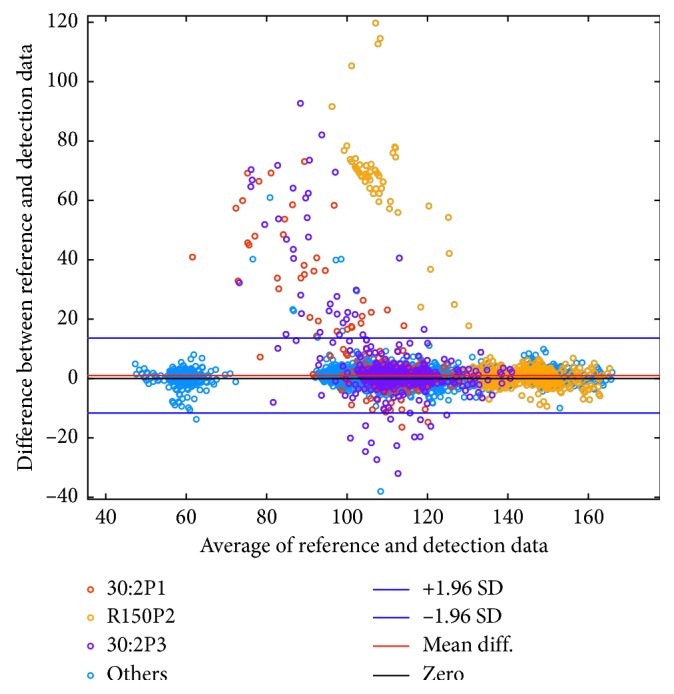
Bland Altman plot to compare reference data from Resusci Anne manikin with detection data from *QCPR cam-app 2.0* for the tests *Smartphone position test*, *Outdoor test*, and *Disturbance test*. All together 11718 compared samples. Different colors are used to differentiate the subtests 30:2P1, R150P2, and 30:2P3 from the rest.

The *Bland Altman* plot in Figure 5 shows the agreement between reference data and detection data for *Smartphone position test*, *Outdoor test*, and the *Disturbance test*. Each analysis in all the test sequences are here included. The subtests with poorer results, *30:2P1*, *R150P2,* and *30:2P3*, is marked with the colors red, yellow, and purple, respectively. The total number of samples in the plot is 11718, and the number of samples with larger deviation than ±10 cpm compared to reference data is 180 (1.53%).

**Figure 6:**
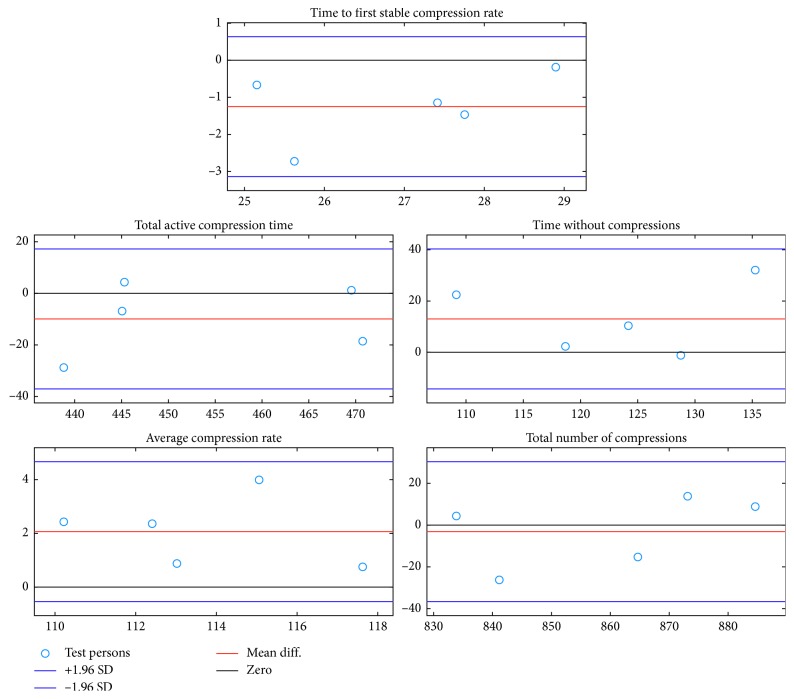
Bland Altman plots of the agreement between the summary parameters calculated from the *QCPR cam-app 2.0* detection data and the summary parameters calculated from the Resusci Anne manikin reference data in the CPR summary report test.

In Figure 6 the *Bland Altman* plots show the agreement between the summary parameters calculated from the detection data and the summary parameters calculated from the reference data in the CPR summary report test.

## 4. Discussion

The results presented in this paper show that the camera in a smartphone can be used to measure chest compression rates and hands-off times under various conditions with good accuracy. Our proposed method allows for real-time feedback to both the bystander and to a dispatcher in real emergencies, which could improve CPR quality.

### 4.1. Challenges

Although the algorithm works well with only a small part of the bystander being visible under low noise situations, we discovered reduced accuracy in two of the sequences where the bystander had long loose hair, compressed with a very high rate and were visible only in a small part of the image frame. In these sequences, the loose hair is sometimes almost the only thing visible in the image frame and *QCPR cam-app 2* interprets this as compression in the rate the visible hair is bouncing in. These two cases explain the yellow samples, in *R150P2*, that caused disagreement in the *Bland Altman* plot, Figure 5. To avoid these false detections the bystander should position the smartphone such that most of the head and shoulders are captured in the image frame.

We also experienced that repetitive random movements during compression pauses could cause the algorithm to detect a false *stable-low* compression rate causing *QCPR cam-app 2* to calculate a longer *TC* and a shorter *TWC*. It could be observed that during compression pauses people often bend towards and away from the patient in a sometime very repetitive movement, and on a few occasion when the bystander had long loose hair, these movements caused the algorithm to interpret the movements as a stable but very low compression rate lasting a minimum of 5 seconds. These false *stable-low* compression rate detections did not occur in the *Random Movement Test* when the test persons were asked to perform all kinds of different tasks that could be carried out before compression starts. Deactivating the *dynamic rate range* could solve this problem, but a consequence of this would be that compressions rates below 70 cpm would not be detected.

The samples that show disagreement between the detections data and the reference data in Figure 5 for subtest *30 : 2P1* (red) and *30 : 2P3* (purple) occurs in the transitions between compression and compression pauses when performing 30:2 and do not significantly affect the visual presentation of the detected signal that is shown to the dispatcher.

### 4.2. Further Work

The proposed system allows the bystander to have both hands free with compression feedback on the smartphone screen visible next to the patient which is different from accelerometer-based smartphone solutions that require the smartphone to be held on the patient's chest or strapped to the bystander's arm [24, 25, 27–30]. This advantage could make the proposed solution suited for real emergencies where the phone is also used as a life line to the emergency unit. Studies comparing the proposed solution with the accelerometer-based solutions in simulated emergencies should be considered.

Testing of *QCPR cam-app 2.0* in simulated real emergencies must be carried out in order to conclude if this method could be suited for real emergencies. In addition, studies with the aim of documenting the usability of the application, safety of the method, and effectiveness on the CPR quality also need to be carried out as suggested by Rumsfeld et al. [16]. If *QCPR cam-app 2.0* shows a well-documented positive effect on the CPR quality, it may be subject to appropriate medical device regulations and made available for clinical use [41, 42].

The detected and stored compression rate signal and the CPR summary report provide further opportunity for evaluation, debriefing and quality improvement of the dispatcher-caller interaction. The stored data and the visual dispatcher feedback system can be used to provide continuing education in T-CPR for dispatchers, as AHA recommends in T-CPR guidelines [43]. In addition, these measurements can provide the EMS arriving at the scene with detailed information about the treatment the patient has received. A feature which records audio and video will be considered integrated in *QCPR cam-app 2*. A possible solution could be to let the recordings be automatically uploaded to a cloud storage when available bandwidth would allow it. Still images, video, and audio could be made available for the dispatcher and allow for a better understanding of the emergency situations. Audio recordings may also be analyzed with respect to chest compression rate and inactivity to further improve measurement accuracy since most dispatcher protocols include prompting and counting loud while compressing on the chest.

The collected data could also be utilized in a machine learning framework providing potential decision support in future systems.

We are currently investigating camera-based methods for measurement of compression *depths* [44]. In future work, we will try to develop a robust depth algorithm that could be implemented together with the proposed method. An implementation of depth measurement would make this solution a complete CPR quality measurement and feedback device. Although the proposed solution's main idea is to assist laypersons in real emergencies, we have also developed a training version of the solution called *TCPR Link*, available on *App Store* and *Google Play* [45, 46] in selected countries. As AHA has announced, CPR feedback devices will also be required to use in all AHA CPR courses by February, 2019 [47].

Studies have also shown that both laypersons and professionals could benefit from objective feedback during CPR. In a study presented by Abella et al. [48], the CPR-certified rescuers performed chest compression rates <80 cpm in 36.9% of the CPR segments included in the study and rates of 100 ± 10 cpm in only 31.4% of the segments, clearly suggesting that CPR-certified rescuers could also benefit from the proposed solution.

### 4.3. Study Limitations

- The validity testing of the *QCPR cam-app 2.0* was assessed with a manikin in a simulated cardiac arrest.
- The *QCPR cam-app 2.0* does not measure chest compression depth.
- The bystanders used in the validity testing were aware of CPR and the *QCPR cam-app 2*.

## 5. Conclusion

Real-time chest compression quality measurement by smartphone camera is feasible for a range of bystanders, compression rates, camera positions, and noise conditions. This technology may be used to measure and improve the quality of telephone CPR and minimize hands-off times.

## Figures and Tables

**Table 1:** Detailed description of the subtests included in the 5 tests performed to both measure the accuracy of QCPR cam-app 2.0's ability to detect the compression rate under various conditions and to evaluate the CPR summary parameters calculated after an ended session.

	Subtest name	Compression rate (cpm)	Duration (s)	Camera position	Lighting	Measures
*Smartphone position test (n*=7)						
RateP1^26^	Normal	60, 100, 120, 150	60 x 4	Pos.1	480 lux	$\mu \bar{E}$, *μP*
D1R110P1^26^	Disturb. person	110	120	Pos.1	480 lux	$\mu \bar{E}$, *μP*
30:2P1	30:2	110	90	Pos.1	480 lux	$\mu \bar{E}$, *μP*
LightP1	Dimmed light	110	60	Pos.1	7 lux	$\mu \bar{E}$, *μP*
R100P2	Small part of image frame (position Change)	100	60	Pos.2	480 lux	$\mu \bar{E}$, *μP*
R150P2	Small part of image frame (position Change)	150	60	Pos.2	480 lux	$\mu \bar{E}$, *μP*
30:2P3	30:2 (position Change)	110	90	Pos.3	480 lux	$\mu \bar{E}$, *μP*
R100P3	Normal (position Change)	100	60	Pos.3	480 lux	$\mu \bar{E}$, *μP*

*Outdoor test (n*=3)						
OCBR110P1	Cloudy with noisy (threes) background	110	60	Pos.1	Cloudy weather	$\mu \bar{E}$, *μP*
OCR110P1	Cloudy with no background	110	60	Pos.1	Cloudy weather	$\mu \bar{E}$, *μP*
OSBR110P1	Sunny with noisy (threes) background	110	60	Pos.1	Sunny weather	$\mu \bar{E}$, *μP*
OSR110P1	Sunny with no background	110	60	Pos.1	Sunny weather	$\mu \bar{E}$, *μP*

*Disturbance test (n*=1)						
D2R110P1	Disturbing person	110	180	Pos.1	Normal indoor	$\mu \bar{E}$, *μP*

*Random movement test (n*=3)						
Ran.MovP1	Random movements	—	150	Pos.1	Normal indoor	*μP*

*CPR summary report test (n*=5)						
CPRsrR110P1	Compressions with pauses	110	580	Pos.1	Normal indoor	*μ*RE_par_

R = rate; P = position; D = disturbance; O = outdoor; B = noisy background; C = cloudy; S = sunny; CPRsr = CPR summary report.

**Table 2:** Detection results for all of the 5 tests included in the experiments.

	$\mu \bar{E}\left(\sigma \bar{E}\right)$ (cpm) (0->)	*μP*(*σP*) (%) (0–100)	*μ*RE_par_(*σ*RE_par_) (%)
*Smartphone position test (n*=7)			
RateP1^26^	1.3 (0.3)	99.7 (0.3)	—
D1R110P1^26^	1.8 (1.3)	99.5 (1.2)	—
30:2P1	4.5 (3.8)	95.9 (3.7)	—
LightP1	1.1 (0.3)	100 (0)	—
R100P2	3.0 (3.4)	98.1 (3.7)	—
R150P2	11.4 (14.9)	89.8 (16.4)	—
30:2P3	3.3 (1.4)	96.0 (2.1)	—
R100P3	1.1 (0.2)	99.9 (0.4)	—

*Outdoor test (n*=3)			
OCBR110P1	1.7 (0.3)	100 (0)	—
OCR110P1	1.5 (0.3)	100 (0)	—
OSBR110P1	1.4 (0.4)	99.7 (0.5)	—
OSR110P1	1.1 (0.4)	100 (0)	—

*Disturbance test (n*=1)			
D2R110P1	5.8	96.0	—

*Random movement test (n*=3)			
Ran.MovP1	—	89.6 (2.5)	—

*CPR summary report test (n*=5)			
CPRsrR110P1	TFSCR	—	6.1 (3.3)
	TC	—	2.8 (2.6)
	TWC	—	10.0 (9.1)
	ACR	—	1.8 (1.2)
	NC	—	1.6 (1.0)
Total (all tests)	2.7 (5.0)	98.0 (5.5)	4.5 (3.6)

The results are given in mean *Average error*, $\mu \bar{E}$, mean *Performance*, *μP*, and mean *Relative error* parameter, *μ*RE_par_. Standard deviations are shown in parenthesis. R = rate; P = position; D = disturbance; O = outdoor; B = noisy background; C = cloudy; S = sunny; CPRsr = CPR summary report.

## Data Availability

The data used in the evaluation of the study are included as supplementary materials in this published article. Our system does not capture and store the videos but only detects, thus the videos cannot be made available.

## Appendix

### Method

This appendix provides a pseudocode description of method for measurement of chest compression rate. More details can be found in [31, 36]. The application is called *TCPR link* and is available on *App Store* [43] and *Google Play* [44].

Let the input, *f*_*l*_(*i*, *j*), represent video frames, *l*, where (*i*, *j*) corresponds to row index *i* and column index *j*. Output is the filtered compression rate measurement, CR_*f*_(*n*), for each 0.5 sec analysis interval, *n*.

  Input: *f*_*l*_(*i*, *j*), Output: CR_*f*_(*n*)

 
**while** receiving image frames **do**

    **Activity measurement:**

(1) *Generating difference frame:*
 **for** All pixels in frame **do**
  **if**|*f*_*l*_(*i*, *j*) − *f*_*l*−1_(*i*, *j*)|<=*ε* **then**
   *g*_*l*_(*i*, *j*)⟵0
  **else**
   *g*_*l*_(*i*, *j*)⟵*f*_*l*_(*i*, *j*) − *f*_*l*−1_(*i*, *j*)
  **end if**
 **end for**
(2) *Dividingg*_*l*_(*i*, *j*)*into non-overlapping blocks and finding the sum of change in region block,R*_*k*_ *over the received frames, L, in the last half second:*
 *S*_*R*_*k*__^*L*^(*n*)⟵∑_*L*−1_^*L*^ *S*_*R*_*k*__(*m*)⟵∑_*L*−1_^*L*^∑_(*i*, *j*)∈*R*_*k*__|*g*_*m*_(*i*, *j*)|
(3) *Marks the blocks with S*_*R*_*k*__^*L*^(*n*)>*than the average block activity*, S_*R*_^*L*^(*n*) *, with an indicator function,I*_*R*_*k*__(*n*)*:*
 **if** *S*_*R*_*k*__^*L*^(*n*)> S_*R*_^*L*^(*n*) **then**
  *I*_*R*_*k*__(*n*)⟵1
 **else**
  *I*_*R*_*k*__(*n*)⟵0
 **end if**
 **if** *ROI*_*established*_=*FALSE* **then**

**Establishing ROI**

(4) *Establishes a temporary ROI:*
 *R*_*k*_ ∈ {*T* − *ROI*_*n*_}  *if*∑_*m*=*n*−3_^*n*^ *I*_*R*_*k*__(*m*) ≥ 3
(5) *Fills block-gaps in the temporary ROI:*
 *R*_*k*_ ∈ {*TF* − *ROI*_*n*_}  *if*  *R*_*k*_ is a gap in a connected object in T-ROI
(6) *Choses the largest connected object,LCO, in the TF-ROI: to be the established ROI.*
 *R*_*k*_ ∈ {*ROI*_*n*_}  *if*  *R*_*k*_ ∈ {*TF* − *ROI*_*LCO*,*n*_}
 *ROI*_*established*_=*TRUE*
 **end if**
 **while** *ROI*_*established*_=*TRUE* **do** for each half second:

**Activity signal from ROI**

(7) *Generate difference signal at time point, l:*
 *d*(*l*)=∑_*R*_*k*_∈*ROI*_*n*__∑_(*i*, *j*)∈*R*_*k*__ *g*(*i*, *j*)

**Frequency analysis**

(8) *STFT is performed on overlapping blocks ofd*(*l*)*, with blocklengthL*_*f*_ *corresponding to 3 sec., updated every 0.5 sec. A sliding Hanning window is used prior to the STFT. The PSD,D*_*n*_(*w*)*, is estimated by the periodogram calculated from the STFT:*
 *D*_*n*_(*w*)=1/*L*_*f*_|ℱ^*M*^{*d*_*hf*_(*l*)}|^2^  *l*=(*n* − 1)*L*_*f*_ : *nL*_*f*_
 where ℱ^*M*^ denotes M point FFT, and *d*_*hf*_(*l*) denotes the Hanning filtered difference signal.

**PSD modelling:**

(9) *Decision tree. Recognizes and handle cases of long loose hair and separate compressions from noise. Relevant frequency range is 40-160 (cpm):*
  Attributes found from *D*_*n*_(*w*):
 (1) Amplitude of the first significant peak, *a*_*p*1_(*n*),
 (2) Amplitude of the second significant peak, *a*_*p*2_(*n*),
 (3) Frequency of the first significant peak, *f*_*p*1_(*n*),
 (4) Frequency of the second significant peak, *f*_*p*2_(*n*) and
 (5) Mean amplitude hight of PSD, *a*_*PSD*_(*n*).
 *CR*(*n*)⟵*decisionTree*(*a*_*p*1_(*n*), *a*_*p*2_(*n*), *f*_*p*1_(*n*), *f*_*p*2_(*n*), *a*_*PSD*_(*n*),

**Post processing**

 *CR*_*f*_(*n*)=*CR*(*n*)
(10) *Short spike/drop removal:*
 **if**|*CR*_*f*_(*n* − 1) − *CR*_*f*_(*n* − 1 − *k*)| < *T*_*sd*1_  ∀*k* ≤ 2**then**
  **if**|*CR*(*n*) − *CR*_*f*_(*n* − 1)| > *T*_*sd*2_ **then**
   *CR*_*f*_(*n*)=*CR*(*n* − 1)
   *i*=*i*+1
   **if** *i*=4**then**
    *CR*_*f*_(*n* − 3 : *n*)=*CR*(*n* − 3 : *n*)
    *i*=0
   **end if**
 **else**
   *i*=0
  **end if**
 **end if**
(11) *Smoothing mean filter:*
 **for** *j*=1 : 3**do**
  *K*=argmax_*J*_ |*CR*_*f*_(*n*) − *CR*_*f*_(*n* − *j*)| < *T*_*mf*_, ∀*j* ≤ *J*
 **end for**
 *CR*_*f*_(*n*)=∑_*k*=0_^*K*^ *a*_*k*_  *CR*_*f*_(*n* − *k*)
 where *a*_*k*_ is the filter coefficients, ∑_*k*=0_^*K*^ *a*_*k*_=1
 and *a*_*j*_=*a*_*i*_  ∀  *i*, *j*.
(12) *Dynamic rate range:*
 *CR*_*drr*_(*n*)=*CR*_*f*_(*n*);
 **if** *CR*_*drr*_(*n*) < 70**then**
  **for** *j*=1 : 10**do**
   *K*=argmax_*J*_ |*CR*_*drr*_(*n*) − *CR*_*drr*_(*n* − *j*)| < *T*_*drr*_, ∀*j* ≤ *J*
  **end for**
  **if** K=10 **then**
   *CR*_*f*_(*n* − 10 : *n*)=*CR*_*drr*_(*n* − 10 : *n*)
  **else**
   *CR*_*f*_(*n*)=0
  **end if**
 **end if**

**ROI update:**

(13) *Add and remove blocks in ROI:*
 **if** *S*_*bo*_*i*__^*L*^(*n*)>0.5· S_*R*_^*L*^(*n*) **then**
  *R*_*bo*,*i*_ ∈ {*ROI*_*n*_}
 **end if**
 **if** *S*_*bi*_*i*__^*L*^(*n*)>0.5· S_*R*_^*L*^(*n*) **then**
  *R*_*bi*,*i*_ ∉ {*ROI*_*n*_}
 **end if**
 where *R*_*bo*,*i*_ denote block i on the outside of the ROI_n_ boundary and *R*_*bi*,*i*_ denote block i inside the ROI_n_
(14) *Freq. analysis if ROI is divided into multiple areas:*
 **if**# of connected areas, *A*_*ROI*_, ∈{*ROI*_*n*_} > 1**then**
  **for** *i*=1 : #  *of*  *A*_*ROI*_ **do**
   Perform step 7, 8 and 9, and
   **if** *CR*_*A*_*ROI*_,*i*_(*n*) is in range of 40–160 cpm **then**
    *A*_*ROI*,*i*_ ∈ {*ROI*_*n*_}
   **else**
    *A*_*ROI*,*i*_ ∉ {*ROI*_*n*_}
   **end if**
  **end for**
 **end if**
 **if**# of *R*_*bi*,*i*_ ∈ {*ROI*_*n*_} < 2**then**
  *ROI*_*established*_=*FALSE*
 **end if**
 **end while**
**end while**

## Supplementary Materials

Supplementary Materials The supplementary material consists data files for each of the five presented experiments. For the first four experiments, the data are presented for each test person as Matlab mat-files containing both the compared reference data and the detection data. For the fifth test, CPR summary report test, the data is presented as ssx-files (reference data) and xls-files (detection data). A readme-file explaining the preprocessing performed prior to the comparison between reference data and detection data is here also included.
